# Emotional eating in healthy individuals and patients with an eating disorder: evidence from psychometric, experimental and naturalistic studies

**DOI:** 10.1017/S0029665120007004

**Published:** 2020-08

**Authors:** Julia Reichenberger, Rebekka Schnepper, Ann-Kathrin Arend, Jens Blechert

**Affiliations:** Department of Psychology, Centre for Cognitive Neuroscience, Paris-Lodron-University of Salzburg, Hellbrunnerstr, 34, 5020 Salzburg, Austria

**Keywords:** Emotions, Binge eating, Ecological momentary assessment, Questionnaire

## Abstract

Emotional eating has traditionally been defined as (over)eating in response to negative emotions. Such overeating can impact general health because of excess energy intake and mental health, due to the risks of developing binge eating. Yet, there is still significant controversy on the validity of the emotional eating concept and several theories compete in explaining its mechanisms. The present paper examines the emotional eating construct by reviewing and integrating recent evidence from psychometric, experimental and naturalistic research. Several psychometric questionnaires are available and some suggest that emotions differ fundamentally in how they affect eating (i.e. overeating, undereating). However, the general validity of such questionnaires in predicting actual food intake in experimental studies is questioned and other eating styles such as restrained eating seem to be better predictors of increased food intake under negative emotions. Also, naturalistic studies, involving the repeated assessment of momentary emotions and eating behaviour in daily life, are split between studies supporting and studies contradicting emotional eating in healthy individuals. Individuals with clinical forms of overeating (i.e. binge eating) consistently show positive relationships between negative emotions and eating in daily life. We will conclude with a summary of the controversies around the emotional eating construct and provide recommendations for future research and treatment development.

## Homeostatic and non-homeostatic influences on eating behaviour

Fulfilling basic human needs such as breathing, sleeping or eating ensures survival. Regarding the latter, in its simplest form, food intake is initiated in states of hunger and energy deficit and terminated upon satiation, thus representing a homeostatic balance of energy intake and expenditure. However, human subjects regularly consume more food than needed and such overeating can lead to negative physiological and psychological health outcomes^(1,2)^. In extreme forms, such overeating is referred to as binge eating, defined as the consumption of an unusual large amount of food in a short time alongside the loss of control (DSM-5^(3)^). Frequent and regular binge eating episodes are a defining criterion for eating disorders such as bulimia nervosa, binge eating disorder, but also the binge–purge subtype of anorexia nervosa.

The prominence of non-homeostatic influences on food intake might be related to the high availability and affordability of palatable and high-energetic foods in nowadays' prosperous societies. A range of factors influence deviations from homeostatic eating, such as social norms, availability of foods, cultural traditions, eating styles/food cravings/food addiction and eating habits^(4)^. The present review will focus on the role of emotions for initiating or modulating eating, as negative emotions have been shown to be one of the most important non-homeostatic reasons for overconsumption^(5,6)^.

## Emotional eating: definition, scope and significance for science and practice

Ice cream after a breakup, potato chips while watching television after a stressful day, chocolates while preparing for an exam; additional food intake in response to sadness and worry (i.e. emotions) is well reflected in folk psychology. In German, the term ‘Kummerspeck’ (‘grief bacon’) relates to the consequences of these phenomena, namely an increase in weight and body fat. Scientifically, emotional eating can be defined as eating in response to negative emotions. This seemingly simple concept has kept research across several disciplines busy in the past four decades. Emotional eating theories have been discussed in social psychology^(7)^, clinical psychology and psychotherapy (patients with an eating disorder^(8)^), nutrition sciences (emotional eating and dieting^(9)^), health psychology, public health (snacking and physical health^(10)^) and metabolic sciences^(11)^, among others. Interest in emotional eating is further fuelled because of its clinical significance in binge eating: patients with eating disorders regularly attribute their binge eating episodes to negative affect^(12)^ and correspondingly, negative affect resembles the most widely reported antecedent of binge eating episodes^(13)^. Due to this high clinical significance, emotional eating research has developed several rivalling families of theories to explain such phenomena.

## Theories of emotional eating

The most widespread emotional eating theories differ in their focus and emphasis on (a) interoception, (b) cognitive processes and (c) learning processes. In the following section, we will briefly introduce one prominent exemplar of each of these theory families.

Psychosomatic theory, exemplifying an interoception-based theory was introduced by Hilde Bruch in 1955^(14)^ to explain psychological factors causal to obesity. Accordingly, individuals with obesity overeat in response to negative emotions because of a lack of interoceptive awareness (e.g. an internal sensation of hunger). Thus, individuals with obesity might confuse physiological arousal related to the emotions with hunger and therefore respond with eating instead of engaging in more functional emotion regulatory strategies. The psychosomatic theory is largely disconfirmed as an account of obesity, while interoception continues to be a fruitful concept in eating behaviour and dieting in particular^(15)^.

Illustrating one of the more cognitive theories, and in opposition to psychosomatic theory, restraint theory was developed^(16)^. The theory states that some individuals who want to lose weight are prone to develop rigid dieting rules (e.g. ‘never eat chocolate’). As a result, even minor violations of such rules can lead to cognitive abandonment of the rule and to overeating (‘what the heck’ effect). Importantly, in the present context, emotions might interfere with the cognitive control needed to uphold such strict diet rules. Restraint eating theory remains central to current emotional eating theorising, as it is pivotal in weight loss dieting.

Regarding learning-based emotional eating theories, the affect regulation model^(17)^ proposes that the rewarding aspects of palatable food intake counter the negative emotions and make such behaviour more likely in the future through the principles of operant conditioning (negative reinforcement). Repeated pairing of negative emotions and eating can further lead to classical conditioning which results in increased motivation to eat in the presence of negative emotions^(18)^.

Several physiological theories have been articulated, owing to the observation that many physiological effects of stress and negative emotions affect key hormones such as cortisol, insulin or glucose but are beyond the scope of this review. Similarly, several nutritional components have been linked with the precursors of neurotransmitters, potentially explaining the mood-alleviating and stress-reducing effects of food intake and the reader is referred to respective review papers^(19,20)^.

## The scope of the present review and the role of the type of emotions

**Fig. 1.**
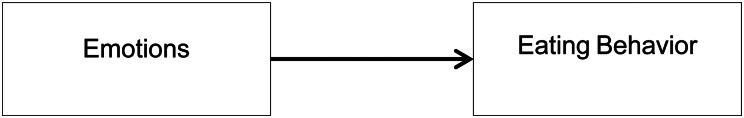
Main effect model of emotional eating.

Irrespective of the underlying theory, the empirical evidence for emotional eating is surprisingly mixed. Hence, we will review the literature from psychometric, experimental and naturalistic studies, focusing on the effect of negative emotions on food intake (see Fig. 1) with particular emphasis on methodological factors that might give rise to this heterogeneity of empirical evidence. We will exclude studies that study the effect of eating or food components on subsequent mood or emotions (instead of the effect of emotions on subsequent eating) as these tap into a different set of theories and are likely less helpful for explaining binge eating and non-homeostatic overeating. We will further exclude studies that explicitly focused on the effect of stress on subsequent eating, due to the unclear relationship of this literature with emotional eating. In addition, we will give particular emphasis to the type and valence of the emotions in question, as this might be an important moderator of how eating is affected (increased or decreased food intake).

## Different types of emotions and individual differences

The idea that specific discrete emotions might differ in their effect on subsequent eating has led to intense research efforts. Macht^(21)^, for example, proposed that negative emotions can both increase or decrease food intake depending on their intensity: high arousal negative emotions such as fear or anger might decrease intake, owing to the physiological influence on metabolism, whereas medium-level negative emotions might increase intake. Relatedly, research started to acknowledge the role of positive emotions for increased food intake^(22–24)^, but the mechanisms involved might be different and will therefore not be covered here in detail.

**Fig. 2.**
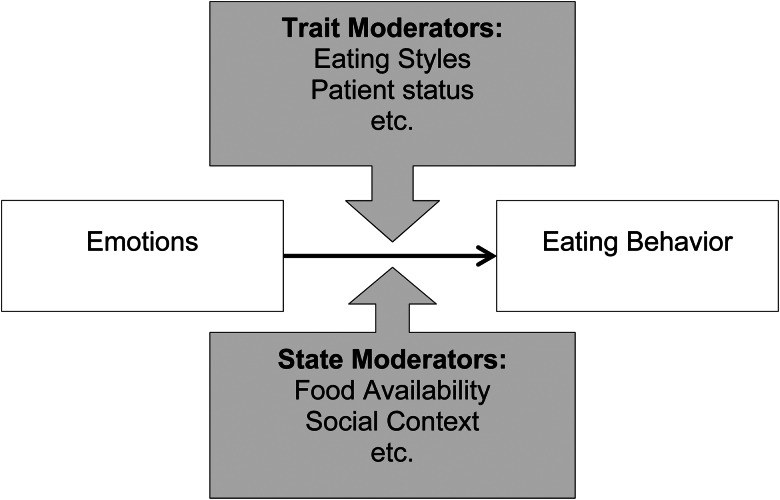
Moderation model of emotional eating.

So far we have conceptualised the relationship between negative emotions and eating as a general and fundamental phenomenon (i.e. main effect model), but there are actually marked individual differences (i.e. moderation model). As can be seen in Fig. 2, several trait and state factors moderate the emotional eating relationship, indicative of inter- and intra-individual differences. To illustrate, previous research reported that trait eating styles such as restrained eating, i.e. a tendency to restrict food intake in order to maintain or lose weight, and emotional eating, i.e. an individual's habitual tendency to eat in response to negative emotions, as well as pathological forms reflected by eating disorders (e.g. bulimia nervosa, binge eating disorder) are likely to show different patterns of emotional eating compared to those scoring low on these eating styles and those without an eating disorder diagnosis. To tap into such inter-individual differences, several psychometric questionnaires have been developed which we will review in the next section. Other factors to consider are contextual or state factors. Easier food availability might make emotional eating more likely, e.g. Zenk *et al*.^(25)^ found that the positive relationship between more daily hassles and more snack-food intake was stronger when foods were easily available. Furthermore, social context might influence emotional eating as the social context might alter emotional experiences and determine whether someone overeats or not^(26,27)^. Similarly, other consummatory behaviour might play a role, in that smoking or excessive alcohol consumption (i.e. unhealthy habits) might be used instead of eating behaviour. To illustrate, we found that in times of high perceived stress, non-smokers report increased food intake whereas smokers decrease their food intake, potentially because they rather rely on smoking instead of eating as a way to cope with the stress^(28)^. Also, emotion regulation might play a role and might affect the emotion–eating link both as trait or state level (thus not displayed in Fig. 2) in that making use of adaptive emotion regulation strategies such as reappraisal or acceptance might dampen the effect of emotions on eating behaviour. To illustrate, Svaldi *et al*.^(29)^ demonstrated that in daily life, the impact of emotions on eating behaviour depends on various emotion regulation strategies.

## Evidence from psychometric research

A range of questionnaires have been developed to measure emotional eating as a trait, personality-like disposition. Questionnaires differ on the types of emotions assessed and the wording of actual eating, desire to eat and eating increase *v.* decrease. One of the most frequently used measures is the Dutch eating behaviour questionnaire^(30)^ measuring the effect of emotions and emotion-related states (including, e.g. boredom) on desire to eat. The three factors eating questionnaire uses emotional eating items on the ‘disinhibition’ subscale^(31)^. Other scales are the emotional eating scale^(32)^, the emotional overeating questionnaire^(33)^, the emotional appetite questionnaire^(34)^ or the positive–negative emotional eating scale^(35)^. The properties of these scales have been reviewed before, e.g. in Bongers and Jansen^(36)^. One of the newer questionnaires is the Salzburg emotional eating scale^(37)^, developed in our workgroup and we will thus briefly review its measurement concept and initial validation data.

The Salzburg emotional eating scale expands the concept of negative emotional eating in mapping the effects of different basic negative and positive emotions on both over- and undereating to more fully represent the relationship between emotions and eating. It includes subscales for happiness, sadness, anger and anxiety and assesses their effects on increased or decreased food intake. Results revealed that participants reported increased eating when experiencing sadness, unchanged eating when being happy and decreased eating when experiencing anger or anxiety^(37)^. This is generally in line with the model by Macht^(21)^ that postulated differences between the basic emotions. Moreover, we found that patients with bulimia nervosa reported increased food intake in response to all three negative emotional subscales, whereas patients with anorexia nervosa reported increased food intake in response to happiness, and decreased food intake to the negative emotional subscales^(38)^, validating the clinical usefulness of the scale and documenting the role of psychopathology as a moderator of the interaction between emotion and eating.

Yet, several researchers have questioned the validity of emotional eating questionnaires in their prediction of actual food intake, both in the laboratory and in daily life^(36,39)^. Evers *et al*.^(40)^ coined the term ‘triple recall bias’ to describe the sources of error in self-reported emotional eating: in order to validly complete such questionnaires, first, a negative emotional state has to be accurately recalled, secondly the respective eating behaviour and thirdly the connection between both. Lastly, respondents ideally aggregate over several such instances to determine a response that is representative to the gross of such situations. Clearly, multiple sources of error are likely to bias the questionnaire scores and might lead to inconsistent effects when actual food intake is assessed as a function of such questionnaire scores. To illustrate, Bongers and Jansen^(36)^ reviewed studies on differences between high and low trait emotional eaters in the laboratory (i.e. food intake in response to a negative compared to a neutral mood condition) and in daily life settings (i.e. food intake in response to daily negative emotions). They found that higher emotional eating questionnaire scores did not consistently predict more eating in the laboratory and daily life. Bongers and Jansen^(36)^ offered several alternative accounts about why some individuals experience their emotions and eating as related. First, self-reported emotional eating might more adequately be interpreted as a more general concept of low self-control and concerns about (over-)eating. Secondly, emotional eating might rather be a retrospective attribution of overeating to negative affect^(41)^, i.e. emotion not being causal for the increased eating but retrospectively ‘constructed’ as a possible reason, or even an excuse for overeating. Thirdly, emotional eaters, when under stress, might overestimate their food intake, despite normal actual intake^(42)^. Thus, individuals with normal consumption misidentify themselves as emotional eaters. Fourthly, self-reported high emotional eaters might be characterised by a generalised learned cue-reactivity in which a variety of cues such as negative and positive emotional states but also the sight and smell of food, the environment one is in or time of day can elicit eating behaviour^(43)^.

## Summary and suggestions for future research: psychometric studies

To summarise, measures for assessing emotional eating vary with regard to the emotions included in the questionnaire (e.g. negative *v.* positive, generic *v.* specific) and the resultant eating behaviour (e.g. tendency to eat *v.* actual food intake), potentially contributing to inconsistent results. In addition, self-reported emotional eating suffers from biases (e.g. recall bias) similar to other subjective assessments^(44)^. Future research might thus profit from comparing various self-report scales (as done in e.g.^(45)^) and explicitly testing their ecological validity (as done in e.g.^(46)^) in addition to doing experimental research under controlled conditions to minimise such biases and enable causal conclusions.

## Experimental studies on emotional eating

Laboratory-based studies provide high control over potentially confounding contextual factors and allow for an objective measure of food intake. Causal effects of emotional state are investigated by using induction of emotions in the laboratory and by assessing subsequent food intake. Mood/emotion induction methods vary from more standardised methods such as exposure to movie excerpts, music or vignettes to more idiosyncratic approaches in which participants recount and imagine recent individual emotional experiences or are exposed to stressful evaluated speech tasks such as in the Trier social stress test (see^(47)^ for more details). Various approaches have been followed also for assessing food intake. The gold standard method is the so-called bogus ‘taste’ test, where participants are asked to give taste ratings of various foods while actual food intake is unobtrusively measured^(48)^. Various factors in the design of a food intake measure need to be considered such as the range and taste quality of offered foods. For example, actual food intake can be assessed in total energy or grams of certain foods offering sweet (e.g. cookies, ice cream, etc.), savoury (e.g. crisps, pretzels, etc.) or both types of foods. In the following, we will review a few exemplar experimental studies to illustrate the laboratory approach to emotional eating.

## Experimental research supporting the validity of emotional eating

Providing support for emotional eating in a laboratory setting, Van Strien *et al*.^(49)^ exposed participants to a negative (via a sad movie in study 1 and a stress task in study 2) and a neutral (via a neutral movie in study 1 and a control task in study 2) mood condition and assessed their subsequent food intake. Trait emotional eating moderated the emotional eating relationship in that high emotional eaters consumed more food on a taste test following the sad movie and the stress task compared to the neutral conditions, whereas low emotional eaters showed the opposite pattern. In contrast to the standardised stressors/induction methods in Van Strien *et al*.^(49)^, in our study, we opted for an idiosyncratic approach to approximate participants' actual real-life stressors^(50)^. To do so, an idiosyncratic interview first explored a recent situation that triggered emotions such as sadness or frustration^(51)^. In the task, participants were then presented with sentences describing this situation, intending to trigger the respective memories and emotions. Interleaved with the sentences, food and object pictures were presented. This setup allowed for the assessment of ratings of momentary desire to eat for each food image instead of actual food intake, alongside recordings of electroencephalography, and other psychophysiological markers of emotion-related food cue reactivity. The key finding was that trait emotional eating moderated the emotional eating relationship in that high emotional eaters increased whereas low emotional eaters decreased their food craving ratings in the negative compared to the neutral mood condition. This was paralleled by a specific pattern of neural activity that indicated that also more implicit response levels were engaged by the task. Note that we opted against a taste test as a dependent variable and measured desire to eat and psychophysiological responses to food cues instead. These responses are sometimes termed ‘food cue reactivity’, and might be less sensitive to the social desirability effects that impact actual food intake in the laboratory.

## Experimental research questioning the validity of emotional eating

Contradicting these results, Braden *et al*.^(45)^ conducted two laboratory-based studies. Study 1 used a mood induction via a sad clip from a drama series and a neutral clip from a nature documentary. Study 2 used a mood induction by a guided imagery exercise to identify and re-experience a recent memory associated with a negative emotion or a neutral route typically taken and assessed food intake in a bogus taste test. The authors revealed that in both studies self-reported emotional eating did not relate to emotional eating in the laboratory. Similarly, Evers *et al*.^(40)^ conducted four studies using different mood induction methods, namely vignettes (study 1), film excerpts (study 2), recall (study 3) and providing false feedback (study 4), to induce negative or neutral/positive emotions. They found no increase in food intake in a bogus taste test after the induction of negative emotions compared to the control conditions in self-described emotional eaters regardless of induction method. Such inconsistencies call for systematic reviews that could try to identify boundary conditions within which current theories make valid predictions (e.g. emotional eating only in certain contexts such as being at home or being alone, or only in certain individuals such as patients with an eating disorder). In addition, the meta-analytic investigation could look at quantitative evidence aggregated across studies while also considering the variation of the studies in moderator analyses.

Cardi *et al*.^(22)^ conducted a meta-analysis on emotional eating in the laboratory which included thirty-three studies with a total of 2491 participants ranging from healthy controls to patients diagnosed with an eating disorder and participants with obesity. They found that overall participants consumed more food under the negative compared to the neutral mood condition (i.e. the main effect). In addition, participant group, mood induction (method as well as the type of mood) and offered food types influenced the strength of the relationship: more food consumption in the negative mood condition was found for participants with pathological eating behaviour (binge eating disorder, subthreshold binge eating disorder and restrained eaters) compared to mentally healthy participants with obesity and healthy controls without obesity. Similarly, Evers *et al*.^(23)^ reported on a meta-analysis including fifty-six studies (twenty-seven of those included in the aforementioned meta-analysis) with a total of 3670 participants ranging from healthy controls to individuals with pathological eating behaviour (i.e. emotional eaters, patients with an eating disorder or participants with obesity). In contrast to Cardi *et al*.^(22)^, Evers *et al*.^(23)^ found no significant overall effect of negative emotion condition on food intake. Again, mood induction method significantly influenced the results in that participants in the social feedback method consumed less food than participants confronted with aversive social materials (movie clips, vignettes, sad stories). The level of restrained eating was again a significant moderator. Restrained eaters consumed a larger amount of food in the negative compared to the neutral mood condition. However, unexpectedly, trait emotional eating did not exhibit a significant moderation effect nor did eating- or weight-related pathology moderate the emotion–food intake relationship as found by Cardi *et al*.^(22)^. Evers *et al*.^(23)^ explained the discrepancy to Cardi *et al*.^(22)^ by (a) additional, new studies, and (b) broader search terms which resulted in a higher number of included studies and (c) by only including studies with a reliable mood induction.

## Summary and suggestions for future research: experimental studies

To summarise, findings from experimental settings are markedly inconsistent, but, in line with the individual differences approach outlined earlier, the investigated sample seems to play an important role (e.g. restrained eaters, patients). The two available meta-analyses agree on the influence of the emotion type/mood induction method, consistent with the idea elaborated earlier that specific emotions differ in their effect on eating. More recently, research has shifted towards more naturalistic assessment methods to circumvent the limitations of laboratory food intake assessment which might be problematic as individuals might alter their eating behaviour because of the heightened self-awareness^(52)^. Furthermore, the highly standardised setting limits possible types of emotions and possible food choices which may not be matched to individual preferences.

## Conducting naturalistic research with regard to emotional eating

To remedy these limitations, research has turned to the assessment of emotional eating in naturalistic, daily life settings using ecological momentary assessment (EMA). EMA is the assessment of daily experiences, behaviour, physiological and psychological status as individuals engage in their natural environment^(53)^. As an advantage, recall biases can be minimised, whereas ecological validity and generalisability can be maximised. Additionally, apart from between-person relationships, EMA studies allow for assessing within-person relationships. This method of assessment seems especially important with regard to eating behaviours as it helps to sample highly dynamic states such as affect and to determine relationships with other dynamic variables such as eating^(54,55)^. EMA studies afford various sampling schemes: signal-contingent sampling involves prompting participants at specific time points whereas in event-contingent sampling participants self-initiate a survey upon the occurrence of specific behaviour (e.g. eating) or situations (e.g. stress). The frequency of daily assessment on signal-contingent sampling balances participant load with the rate at which the phenomena of interest change (mood, eating or hunger). The naturalistic context allows EMA assessment schemes to measure eating behaviour more broadly (see also^(56)^): information can be obtained on desire to eat ratings^(57)^, snacking^(25)^, specific food item intake^(58)^, energy density of meals^(59–61)^ but also loss of control over eating^(62)^ or clinical binge eating episodes^(63)^.

## Evidence from naturalistic research

### Healthy individuals

Haedt-Matt *et al*.^(64)^ asked 239 female twins from a community-based sample about their affect and emotional eating urges once daily for 45 consecutive days and showed that higher negative affect was concurrently associated with higher emotional eating urges, providing support for emotional eating in naturalistic EMA settings. Other studies in mostly healthy individuals showed that negative affect related to greater binge eating^(65,66)^, more consumption of comfort food^(67)^ and more consumption of meat/protein^(68)^. While these studies support the main effect model where emotions are linked with eating regardless of another person-level moderator, we recently found support for an individual difference (i.e. moderation) model: our EMA study in fifty-nine participants involved five daily signals for 10 days on current negative emotional state and eating behaviour^(69)^. We aimed at the hedonic component of eating, thus we asked participants to report on the extent to which they ate their last meals out of taste (as opposed to hunger). Results revealed that trait emotional eating moderated the emotional eating relationship in daily life in that low emotional eaters decreased their taste-eating with increasing negative emotions, whereas high emotional eaters increased their taste-eating with increasing negative emotions.

Contradicting these findings, Adriaanse *et al*.^(39)^ conducted two studies asking 151 and 184 participants once daily for 7 days to report the amount of their healthy and unhealthy snacks. Whereas trait emotional eating status did not explain unhealthy snacking, self-reported habitual snacking and dietary restraint did explain unhealthy snacking. Various other studies showed that negative affect did not relate to subsequent unhealthy eating^(70)^, snack intake^(25)^ or subclinical pathological eating behaviour such as eating large amounts of food^(71)^, or even related to decreased subsequent energy consumption^(72)^ in healthy individuals.

### Individuals with an eating disorder

Reviewing thirty-six previous naturalistic studies in a total of 968 individuals with an eating disorder, Haedt-Matt and Keel^(73)^ showed that negative affect precedes binge eating, although post-binge negative affect was even increased. In more detail, negative affect was greater prior to binge eating compared to general levels of negative affect or prior to other regular eating episodes. Diagnosis (bulimia nervosa *v.* binge eating disorder) accounted for a significant amount of variability in that the relationships were smaller in individuals with bulimia nervosa compared to those with binge eating disorder. Moreover, assessment parameters such as the sampling scheme (signal- or event-based sampling), length and frequency of EMA assessment as well as provisions of binge eating definitions influenced the magnitude of the relationship between affect and binge eating. The majority of EMA studies in patients with an eating disorder demonstrated a positive relationship between negative emotions and binge eating episodes, including also subcomponents such as over- or loss of control eating^(29,62,74–80)^. EMA research also investigated the types of negative emotions that precede binge eating: Becker *et al*.^(81)^ showed that emotions high on negative valence, arousal and avoidance-relation precede a binge eating episode. In contrast, Berg *et al*.^(82,83)^ emphasised the role of distinct emotions such as fear, hostility, sadness, but especially guilt in preceding binge eating episodes.

## Summary and suggestions for future research: naturalistic studies

To summarise, in healthy individuals, large variability arises, potentially driven by the various assessment strategies for measuring eating behaviour in naturalistic studies. Additionally, lack of standardisation and methodological gold standards in naturalistic studies hinder consensus. Also in children and adolescents, the influence of negative emotions on eating behaviour and dietary intake in daily life remains inconclusive and revealed mixed results (for review see^(84)^). However, a systematic review of the evidence regarding emotional eating in healthy adults in daily life remains a worthwhile future direction. In contrast to findings in healthy individuals, results in individuals with an eating disorder seem quite consistent.

Given the complex relationship between emotion and eating behaviour variables as well as their moderators, new statistical avenues (e.g. machine learning approach) are needed that do not assume the linearity of tested variables. Also, as emotions might impact eating with some delay (e.g. next meal or within the whole day), statistical methods with variable time lags might be needed. In the same vein, contextual and situational factors (e.g. eating alone *v.* in company, food availability) might influence daily emotional eating because of their broader variability in naturalistic settings. Furthermore, subjective and objective data not always correspond so that the integration of objective methods that more accurately characterise participant's emotions and eating behaviour in daily life might be a fruitful future direction (e.g.^(85)^). To illustrate, there are attempts to sense emotional states from heart rate variability readouts^(86)^ or voice audio recordings^(87)^. Similarly, objective food intake can be assisted by obtaining the pictures of the food eaten, food lists^(88)^ or barcode scanning^(89)^, or can be approached by electromyography of swallowing or chewing behaviour^(90)^ or bite counters^(91,92)^.

## General discussion/future directions

**Fig. 3.**
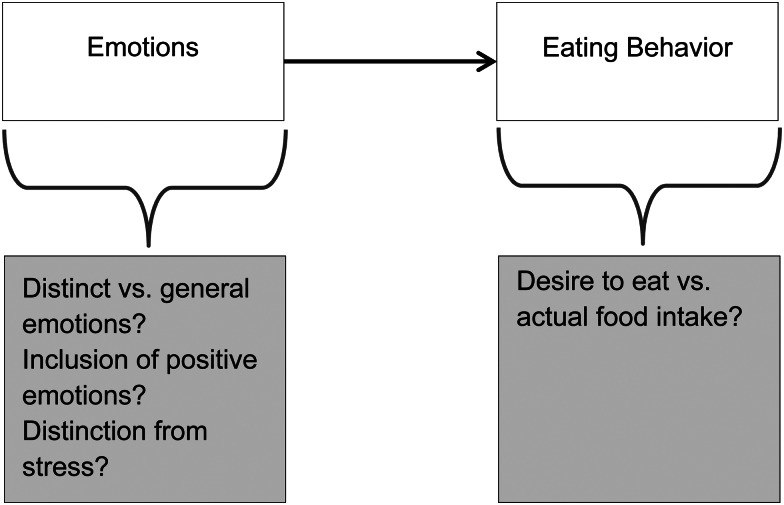
Current controversies and future directions.

To conclude, several controversies characterise the literature reviewed earlier. Open questions relate to the type and intensity of emotions that are assumed to cause changes in eating behaviour (see Fig. 3). A general negative affect model would assume that all negative emotions employ similar mechanisms in driving the need for relief and hedonic improvement. A specific emotion model, by contrast would have to distinguish several specific emotion–eating relationships, one for each specific emotion. In addition, it would be worthwhile to consider the inclusion of positive emotions (e.g. see^(24,93)^) into a broader definition of emotional eating. Another open question is a clearer separation of emotions and stress in their impact on eating behaviour (see^(94)^ for a reasoning on that aspect). Similarly, one has to be aware that eating behaviour can be measured via food craving (i.e. a desire to eat) *v.* actual food and energy intake likely resulting in different results.

A bundle of potential trait and state moderators have been outlined earlier resulting in a complex emotional eating relationship. Consolidation on a theoretical and practical ground might be helpful to further specify the emotional eating construct and aid in testing different theories or mechanisms (outlined earlier) against each other (e.g. for clarifying the role of trait emotional eating *v.* trait restrained eating and related affect regulation theory *v.* restraint theory).

Fruitful future directions show that the combination of various study types might be especially helpful. To illustrate, Smith *et al*.^(95)^ examined laboratory-assessed impulsivity in combination with daily life relationships of negative affect and binge eating and revealed that greater delay discounting strengthened the relationship between negative affect and binge eating. Similarly, Wonderlich *et al*.^(96)^ showed that neural responses to food cues moderate the relationship between negative affect and binge eating in daily life. By combining psychometric, experimental and naturalistic settings, the respective design strengths (experimental research, internal validity; naturalistic setting, external validity) can be combined.

## Clinical implications

Based on the literature reviewed earlier, interventions using cognitive-behaviour therapy, especially emotion regulation interventions might be fruitful in reducing negative affect, which might in turn reduce the likelihood of overconsumption or bingeing. Recently, research started to use the induction of positive emotions as the method to decrease the likelihood of binge eating and promising results have been obtained. To illustrate, Cardi *et al*.^(97)^ induced positive emotions in individuals with bulimia nervosa and binge eating disorder and found that these individuals exhibited less negative emotions and consumed less food in a subsequent taste test compared to a neutral condition (similar results obtained by Sproesser *et al*.^(98)^ in stress eating).

Progress in basic research is currently not paralleled by a corresponding progress in intervention development. Thus, while established guidelines for treating emotional eating in eating and weight disorders exist, hardly any innovative non-face-to-face interventions exist. Only recently have researchers proposed to use online interventions or smartphone-based interventions in daily life, lowering the threshold for treatment engagement. To illustrate, the so-called just-in-time adaptive interventions^(99)^ use subjectively and objectively derived data from several state variables (e.g. current emotions, social context) to detect an optimal time point for sending brief therapeutic text messages, potentially adapted to the participant (e.g. with or without eating disorder). Hence, future research on the construct of emotional eating might pave the way towards personalised treatments for eating and weight disorders.
